# The Impact of Contemporary Antiretroviral Drugs on Atherosclerosis and Its Complications in People Living With HIV: A Systematic Review

**DOI:** 10.7759/cureus.47730

**Published:** 2023-10-26

**Authors:** Abhijith C Vemulapalli, Abanob A Elias, Monica D Yerramsetti, Olawale O Olanisa, Payal Jain, Qasim S Khan, Samia R Butt

**Affiliations:** 1 Internal Medicine, California Institute of Behavioral Neurosciences & Psychology, Fairfield, USA; 2 General Practice, California Institute of Behavioral Neurosciences & Psychology, Fairfield, USA

**Keywords:** human immunodeficiency virus (hiv) infection, risk factors of atherosclerosis, hiv protease inhibitors, hiv integrase strand transfer inhibitors, people living with hiv (plhiv), major adverse cardiovascular event, antiretroviral drugs

## Abstract

With the advent of modern antiretroviral therapy (ART), human immunodeficiency virus (HIV) infection has been modified into a chronic manageable condition, prolonging the lifespan of people living with HIV (PLHIV). This has resulted in an increased non-AIDS-related morbidity in the HIV-infected population. Our aim is to study the role of contemporary ART in tackling the risk of atherosclerosis and cardiovascular disease (CVD) in PLHIV. We searched through the databases of PubMed, PubMed Central, and Cochrane Library for pertinent articles using the medical subject headings (MeSH) “HIV infection”, “Atherosclerosis”, and “Antiretroviral agents”. The articles published in the past five years were retrieved, screened for relevance, and assessed for quality before being included in the review. This review was performed following the PRISMA 2020 guidelines. The results indicate that the incidence of dyslipidemia with integrase strand transfer inhibitors (INSTIs) is greater than with non-nucleoside reverse transcriptase inhibitors (NNRTIs) and lesser than with protease inhibitors (PIs). INSTIs are indispensably associated with weight gain and obesity. High triglyceride (TG) and oxidized low-density lipoproteins to low-density lipoproteins (oxLDL/LDL) ratio levels and low high-density lipoprotein (HDL) levels are seen in patients taking PIs. A higher incidence of hypertension and metabolic syndrome (MetS) was noticed with INSTIs compared to NNRTIs. PI intake for >5 years increases the risk of subclinical atherosclerosis. Increased risk of myocardial infarction with INSTIs was observed in a study, while another study reported decreased risk. HIV infection independently increases the risk for atherosclerosis and CVD. Although contemporary ART decreases this enhanced risk, it inherently increases the risk for abnormal lipid profile, MetS, weight gain, and obesity. Further research into the risk of atherosclerosis and CVD with newer ART drugs is essential for decoding the underlying mechanisms and preventing adverse cardiac outcomes in PLHIV.

## Introduction and background

Human immunodeficiency virus (HIV) infection remains a major global public health issue, with 1.3 million people acquiring HIV in 2022 and an estimated 39.0 million people living with HIV at the end of 2022 [1]. Having claimed 40.4 million lives so far, 630,000 in 2022 alone, transmission continues in all countries globally [1]. However, with access to effective HIV prevention, early diagnosis, treatment, and care, HIV infection has become a manageable, long-term/life-long health condition, enabling people living with HIV (PLHIV) to lead protracted, healthy lives - “The Graying of the HIV Epidemic” [1].

**Figure 1:**
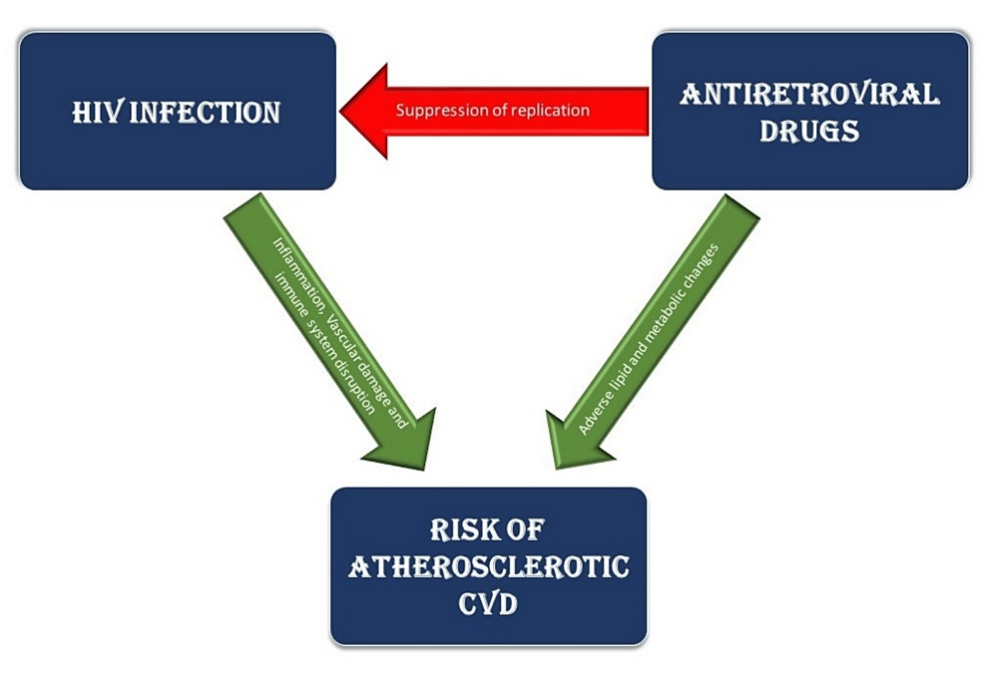
Correlation Between HIV Infection, Antiretroviral Drugs, and Atherosclerotic CVD Risk HIV: Human immunodeficiency virus; CVD: cardiovascular disease This figure is the author's own creation.

In the wake of modern antiretroviral drugs, the importance of acquired immunodeficiency syndrome (AIDS)-related comorbidities and deaths in PLHIV is waning and the significance of cardiovascular disease (CVD) in this respect is rising [2]. It has been well-documented that HIV infection is an independent risk factor for coronary artery disease [2-4]. The risk of CVD in PLHIV is a result of the coaction of traditional CVD risk factors, risk from HIV infection, and the adverse effects of certain antiretroviral drugs [5,6]. The increased risk of CVD, including myocardial infarction (MI) and stroke, is 2.2-fold compared to the general population [7]. This increased risk related to HIV infection could be due to viremia and the associated chronic inflammation and immune dysregulation. Though the initial antiretroviral drugs aided in reducing the viral load and restoring the immune system function to an extent, they came with significant adverse effects [8]. The relation between HIV infection, antiretroviral therapy (ART) drugs, and atherosclerosis risk is depicted in Figure 1. In this new “test-and-treat” era, with expanded access and usage of contemporary ART drugs, the question about their efficacy and safety arises. The adverse metabolic effects of contemporary antiretroviral drugs have been studied to a decent extent, but there is limited knowledge available about their contribution to atherosclerotic cardiovascular disease (ASCVD).

Research question

In this review, we aim to highlight the effects of newer ART drugs on the risk factors and complications of atherosclerosis in PLHIV. This includes the effects of integrase strand transfer inhibitors (INSTIs) and protease inhibitors (PIs) on the lipid and metabolic parameters of HIV-infected patients, along with their influence on the risk for CVD.

Clinical implications

A better understanding of the cardiovascular safety of the newer drugs helps establish better protocols and guidelines for the prescription of these drugs in PLHIV. Avoidance of drugs associated with higher rates of adverse effects in at-risk patients can benefit them by reducing the need for further medication to treat the risk factors or comorbidities, preventing the occurrence of undesirable adverse events, and improving the overall quality of life.

## Review

Methods

Methodology and Search Strategy

The methods and results reported in our systematic review are as per the Preferred Reporting Items for Systematic Reviews and Meta-Analysis (PRISMA) 2020 guidelines following our screening and selection [9].

We searched through PubMed, PubMed Central, and Cochrane Library for the most relevant original studies and reviews using medical subject headings (MeSH), keywords, and Booleans. The search strategies used in different databases and the number of articles identified are presented in Table 1.

**Table 1:** Databases and the Respective Search Strategies Used HIV: Human immunodeficiency virus; HTLV-III: human T-lymphotropic virus type III

Database Used	Search Strategy	Number of Articles Identified
PubMed	(( "HIV Infections/complications"[Majr] OR "HIV Infections/drug therapy"[Majr] OR "HIV Infections/immunology"[Majr] OR "HIV Infections/mortality"[Majr] OR "HIV Infections/prevention and control"[Majr] OR "HIV Infections/therapy"[Majr] )) OR ( "HIV Infections/complications"[Mesh:NoExp] OR "HIV Infections/drug therapy"[Mesh:NoExp] OR "HIV Infections/immunology"[Mesh:NoExp] OR "HIV Infections/mortality"[Mesh:NoExp] OR "HIV Infections/prevention and control"[Mesh:NoExp] OR "HIV Infections/therapy"[Mesh:NoExp] ) AND (( "Atherosclerosis/chemically induced"[Majr] OR "Atherosclerosis/complications"[Majr] OR "Atherosclerosis/drug therapy"[Majr] OR "Atherosclerosis/etiology"[Majr] OR "Atherosclerosis/immunology"[Majr] OR "Atherosclerosis/mortality"[Majr] OR "Atherosclerosis/physiopathology"[Majr] OR "Atherosclerosis/prevention and control"[Majr] OR "Atherosclerosis/therapy"[Majr] OR "Atherosclerosis/virology"[Majr] )) OR ( "Atherosclerosis/chemically induced"[Mesh:NoExp] OR "Atherosclerosis/complications"[Mesh:NoExp] OR "Atherosclerosis/drug therapy"[Mesh:NoExp] OR "Atherosclerosis/etiology"[Mesh:NoExp] OR "Atherosclerosis/immunology"[Mesh:NoExp] OR "Atherosclerosis/mortality"[Mesh:NoExp] OR "Atherosclerosis/physiopathology"[Mesh:NoExp] OR "Atherosclerosis/prevention and control"[Mesh:NoExp] OR "Atherosclerosis/therapy"[Mesh:NoExp] OR "Atherosclerosis/virology"[Mesh:NoExp] ) AND (( "Anti-Retroviral Agents/adverse effects"[Majr:NoExp] OR "Anti-Retroviral Agents/classification"[Majr:NoExp] OR "Anti-Retroviral Agents/therapeutic use"[Majr:NoExp] OR "Anti-Retroviral Agents/toxicity"[Majr:NoExp] )) OR ( "Anti-Retroviral Agents/adverse effects"[Mesh:NoExp] OR "Anti-Retroviral Agents/classification"[Mesh:NoExp] OR "Anti-Retroviral Agents/therapeutic use"[Mesh:NoExp] OR "Anti-Retroviral Agents/toxicity"[Mesh:NoExp] )	10,758
PubMed Central	( "Atherosclerosis/complications"[Majr] OR "Atherosclerosis/drug therapy"[Majr] OR "Atherosclerosis/etiology"[Majr] OR "Atherosclerosis/microbiology"[Majr] OR "Atherosclerosis/prevention and control"[Majr] OR "Atherosclerosis/therapy"[Majr] OR "Atherosclerosis/virology"[Majr] ) AND ( "HIV Infections/classification"[Majr] OR "HIV Infections/complications"[Majr] OR "HIV Infections/drug therapy"[Majr] OR "HIV Infections/immunology"[Majr] OR "HIV Infections/mortality"[Majr] OR "HIV Infections/prevention and control"[Majr] OR "HIV Infections/therapy"[Majr] ) AND ( "Anti-Retroviral Agents/adverse effects"[Majr] OR "Anti-Retroviral Agents/analysis"[Majr] OR "Anti-Retroviral Agents/classification"[Majr] OR "Anti-Retroviral Agents/therapeutic use"[Majr] OR "Anti-Retroviral Agents/toxicity"[Majr] )	9
Cochrane Library	(HIV infection OR HTLV-III infection) AND (Atherosclerosis OR Atheroma OR Atherogenesis OR (Atherosclerotic plaque)) AND (Antiretroviral agents)	41

The articles were then screened to highlight those most relevant to the research question based on the title and abstract and selected per the inclusion/exclusion criteria.

Inclusion and Exclusion Criteria

The selection preference was systematic reviews, meta-analyses, randomized controlled trials (RCTs), and observational studies published from 2018 to 2023. All the articles selected were peer-reviewed and published in the English language. Grey literature was excluded. Reviews without a clear methods section, animal studies, in-vivo, and in-vitro studies were also excluded. Our eligibility criteria were built on the population, intervention, comparison, and outcomes (PICO) model. The inclusion and exclusion criteria are disclosed in Table 2.

**Table 2:** Eligibility Criteria Used for Study Selection HIV: Human immunodeficiency virus; INSTIs: integrase strand transfer inhibitors; PIs: protease inhibitors; AIDS: acquired immunodeficiency syndrome; ART: antiretroviral therapy

Inclusion Criteria	Exclusion Criteria
Studies involving adult patients with HIV infection (age ≥18 years) taking antiretroviral drugs belonging to the drug classes of INSTIs or PIs. Articles published within the last five years. Patients from any geographical region of the world. Articles discussing the effects of INSTIs and/or PIs on atherosclerosis, its risk factors, or its cardiac complications.	Studies involving children (age <18 years), pregnant patients with HIV, patients with perinatally acquired HIV, HIV patients with opportunistic infections or AIDS-defining illnesses, or HIV patients with a history of coronary heart disease or stroke. Studies that have not specified the class of ART used by the participants. Studies with a sample population size of less than 100. Narrative reviews, editorials, animal studies, in-vivo and in-vitro studies. Articles on guidelines.

Three separate authors extracted and reviewed the essential data independently. In case of disagreements, the researchers discussed the conformity of the data to the eligibility criteria to reach a consensus. A fourth researcher was consulted for objectivity if a decision could not be made.

Critical Appraisal of Studies

All the studies selected for the review were critically appraised using the appropriate quality assessment tool. There are seven cohort studies, seven cross-sectional studies, and one RCT included in this review. We used the respective Joanna Briggs Institute (JBI) critical appraisal checklist for the quality check of cohort studies and cross-sectional studies [10]. We used the Cochrane risk of bias assessment tool for the RCT [11]. The quality assessment of the included studies has been presented in Tables 3-5.

**Table 3:** Quality Assessment of the Cohort Studies JBI: Joanna Briggs institute; Y: Yes; N: No; UC: Unclear

JBI Critical Appraisal Checklist for Cohort Studies											
Article	Question 1	Question 2	Question 3	Question 4	Question 5	Question 6	Question 7	Question 8	Question 9	Question 10	Question 11
Rebeiro et al., 2023 [12]	Y	Y	Y	Y	Y	Y	Y	UC	Y	Y	Y
Byonanebye et al., 2022 [13]	Y	Y	Y	Y	Y	Y	Y	Y	UC	Y	Y
Pantazis et al., 2022 [14]	Y	Y	Y	Y	Y	N	Y	Y	Y	-	Y
Zhang et al., 2022 [15]	Y	Y	Y	Y	Y	N	Y	UC	Y	-	Y
Byonanebye et al., 2021 [16]	Y	Y	Y	Y	Y	Y	Y	Y	Y	-	Y
O’Halloran et al., 2020 [17]	Y	Y	Y	Y	Y	Y	Y	UC	UC	N	Y
Bakal et al., 2018 [18]	Y	Y	Y	Y	Y	Y	N	Y	UC	Y	Y

**Table 4:** Quality Assessment of the Cross-Sectional Studies JBI: Joanna Briggs institute; Y: Yes; N: No; UC: Unclear

JBI Critical Appraisal Checklist for Cross-sectional Studies								
Article	Question 1	Question 2	Question 3	Question 4	Question 5	Question 6	Question 7	Question 8
Hamooya et al., 2021 [19]	Y	Y	Y	UC	Y	Y	Y	Y
Tiarukkitsagul et al., 2021 [20]	Y	Y	UC	UC	Y	Y	Y	Y
Serrão et al., 2019 [21]	Y	Y	Y	UC	Y	Y	UC	Y
Bastard et al., 2019 [22]	N	Y	Y	Y	Y	Y	Y	Y
Agu et al., 2019 [23]	Y	Y	Y	Y	Y	UC	Y	Y
Calza et al., 2019 [24]	Y	Y	Y	UC	Y	Y	Y	Y
Alikhani et al., 2019 [25]	Y	Y	Y	UC	Y	Y	UC	Y

**Table 5:** Quality Assessment of the RCT RCT: Randomized controlled trial

Cochrane Risk of Bias Tool for RCTs						
Article	Domain 1	Domain 2	Domain 3	Domain 4	Domain 5	Overall risk of bias
Llibre et al., 2022 [26]	Low risk	Low risk	Low risk	Low risk	Low risk	Low risk

Results

Literature Search and Study Selection

**Figure 2:**
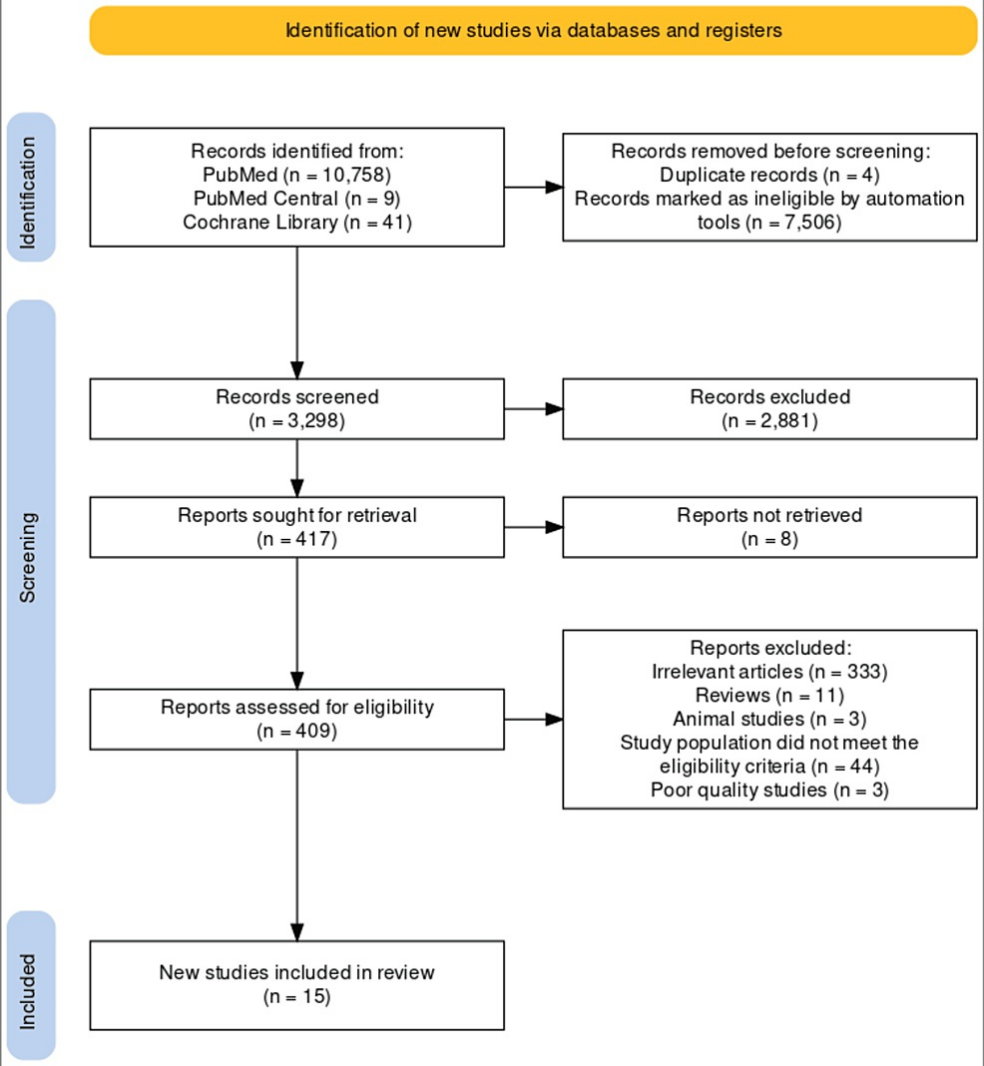
PRISMA Flow Diagram Showing the Process of Study Selection PRISMA: Preferred Reporting Items for Systematic Reviews and Meta-Analysis

The PubMed MeSH search strategy generated 10,758 articles. The search in PubMed Central generated nine articles. The search through the Cochrane Library revealed 41 trials. Four articles were excluded due to duplication. After filtering to include the articles published in the last five years only, we had 3,298 articles. Upon screening through the titles and abstracts of the articles, we obtained 73 articles. Full-text assessment of these articles for the eligibility criteria and risk of bias finally provided us with the 15 articles included in this review. Our PRISMA flow diagram is shown in Figure 2 [9].

Basic Characteristics of the Reviewed Articles

Tables 6-8 show the characteristics of the cohort, cross-sectional, and RCT studies included in our systematic review respectively.

**Table 6:** A Brief Overview of the Cohort Studies INSTIs: Integrase strand transfer inhibitors; MI: myocardial infarction; CHF: congestive heart failure; ART: antiretroviral therapy; NNRTIs: non-nucleoside reverse transcriptase inhibitors; PIs: protease inhibitors; BMI: body mass index; DTG: dolutegravir; RAL: raltegravir; EVG/c: elvitegravir/cobicistat; PLHIV: people living with HIV; TG: triglyceride; MACEs: major adverse cardiac events; PCI: percutaneous coronary intervention

Article	Study Population Size	Outcome of Interest	Study Findings	Conclusion
Rebeiro et al., 2023 [12]	INSTI cohort: 10,115	Incidence of cardiometabolic outcomes in INSTI vs. non-INSTI cohorts	MI, CHF, and lipid disorders are more likely in the INSTI group	INSTI group is associated with an increased risk of cardiometabolic outcomes like MI and lipid disorders
	Non-INSTI cohort: 3961			
Byonanebye et al., 2022 [13]	ART-experienced: 2486	Incidence of hypertension with INSTIs, PIs, and NNRTIs	Higher incidence rate with INSTIs than NNRTIs. PIs and INSTIs had similar incidence rates	Hypertension risk with INSTIs is similar to PIs but greater than NNRTIs
	ART-naïve: 2120		Even higher incidence rate with INSTIs than NNRTIs. PIs and INSTIs had similar incidence rates	
Pantazis et al., 2022 [14]	982	Changes in BMI with INSTIs (DTG vs. RAL vs. EVG), PIs, and NNRTIs	BMI levels increased within all groups with the fastest change in the INSTI group and the slowest change in the NNRTI group	Increased prevalence of obesity in PLHIV with INSTI use, especially with DTG and RAL compared to EVG
Zhang et al., 2022 [15]	112	Lipid profile changes in the hypertriglyceridemia and non-hypertriglyceridemia subgroups of initial PI and NNRTIs to PI groups	Higher TG levels in both groups.	PI-based regimen bolstered the NNRTIs-related hypertriglyceridemia. NNRTIs to PI group individuals with normal TG levels at baseline may sustain them.
Byonanebye et al., 2021 [16]	4577	Incidence of dyslipidemia with INSTIs (DTG vs. RAL vs. EVG), PIs, and NNRTIs	INSTIs had an incidence rate of 29% lower than PIs and 35% higher than NNRTIs	Dyslipidemia was less common with INSTIs than with PIs. More common with EVG/c and RAL than DTG
O’Halloran et al., 2020 [17]	20,242	Occurrence of the MACEs – MI, Stroke, PCI, coronary artery bypass	INSTIs were associated with a lower risk of MACEs compared to other drug classes	INSTIs have reduced the risk of MI and stroke
Bakal et al., 2018 [18]	Baseline analysis: 1794	Prevalence of obesity at ART initiation	Prevalence increased over the study period	The prevalence of obesity in PLHIV is increasing
	Longitudinal analysis: 1567	Incidence of obesity after ART	The use of an INSTI-based regimen is one of the factors associated with obesity development	INSTIs are associated with weight gain and obesity.

**Table 7:** A Brief Overview of the Cross-sectional Studies MetS: Metabolic syndrome; ART: antiretroviral therapy; DTG: dolutegravir; NNRTIs: non-nucleoside reverse transcriptase inhibitors; CVD: cardiovascular disease; ASCVD: atherosclerotic cardiovascular disease; NARCs: non-AIDS-related comorbidities; HIV: human immunodeficiency virus; PIs: protease inhibitors; HDL: high-density lipoprotein; LDL: low-density lipoprotein; oxLDL: oxidized LDL; PAD: peripheral arterial disease; ABI: ankle-brachial index; WHR: waist-to-hip ratio; PLHIV: people living with HIV; AZT: zidovudine (AZT); d4T: stavudine (d4T); RAL: raltegravir (RAL); FMR: fat mass ratio

Article	Study Population Size	Outcome of Interest	Study Findings	Conclusion
Hamooya et al., 2021 [19]	1108	Variables associated with MetS in ART-experienced patients	DTG-based regimen had twice the risk of developing MetS than the NNRTI-based regimen	MetS increases the CVD risk by 75%. Mechanisms underlying ART-associated MetS need further study
Tiarukkitsagul et al., 2021 [20]	460	10-year ASCVD risk with first-line ART vs. second-line ART	Median 10-year ASCVD risk was significantly higher with second-line ART than with first-line ART	Second-line ART is an independent indicator of intermediate to high 10-year ASCVD risk in addition to traditional CVD risk factors
Serrão et al., 2019 [21]	401	Factors associated with NARC in older HIV patients	Hypercholesterolemia followed by arterial hypertension was the most common NARC.	NARC rates were comparable to that of the general population. Age and HIV infection duration were positively related to NARCs
Bastard et al., 2019 [22]	352	Correlation between metabolic changes from long-term ART use and the markers of inflammation and oxidative stress	Patients taking PIs had lower levels of HDL and higher levels of TGs and oxLDL/LDL ratio	Biomarkers of oxidative stress were independently associated with high levels of metabolic comorbidities (Diabetes, LDL-related and atherogenic dyslipidemias)
Agu et al., 2019 [23]	Infected study group: 150	Prevalence of PAD in the study vs. control groups as measured primarily by ABI	No difference in PAD prevalence between the study and control groups. Significant negative correlation between duration of ART and ABI	Higher WHR with NNRTIs than boosted PIs. Elevated dyslipidemia and PAD risk with longer duration of ART
	Uninfected control group: 50			
Calza et al., 2019 [24]	188	Association between vitamin D levels and atherosclerosis in PLHIV	Vitamin D deficiency accelerates atherosclerosis in PLHIV	PI exposure and hypovitaminosis D increase the risk of atherosclerosis in PLHIV
Alikhani et al., 2019 [25]	166	Prevalence and risk of lipodystrophy with ART exposure	AZT, d4T, and RAL are independently associated with an increase in FMR	Lipodystrophy with RAL in this study could be due to reverse causality

**Table 8:** A Brief Overview of the RCT RCT: Randomized controlled trial; CAR: continued antiretroviral regimen; DTG: dolutegravir; RPV: rilpivirine (RPV); FABP: fatty acid-binding protein; sVCAM: soluble vascular cell adhesion molecule

Article	Study Population Size	Outcome of Interest	Study Findings	Conclusion
Llibre et al., 2022 [26]	Early-switch group: 513	Changes in biomarkers of inflammation and atherogenesis after switching to DTG+RPV regimen from CAR	Over 48 weeks, a decrease in FABP-2 favoring DTG+RPV regimen was observed. Over 148 weeks, FABP-2 and sVCAM-1 had continued reductions, while D-dimer showed incongruous increases between early- and late-switch groups	No dependable change patterns post-switch to DTG+RPV were observed over 48 or 148 weeks implying no correlation between the two-drug regimen and increased atherogenesis
	CAR group: 511; Late-switch group: 477			

Definitions

Dyslipidemia was defined as random total cholesterol (TC) >240 mg/dL and/or high-density lipoprotein (HDL) <35 mg/dL, triglycerides (TGs) >200 mg/dL, and/or the initiation of lipid-lowering therapy [16] and/or TG/HDL ratio ≥3.5 in men and ≥2.5 in women [22].

General obesity was defined as body mass index (BMI) ≥30 Kg/m² or waist circumference of ≥80 cm in women and ≥94 cm in men or waist-to-hip ratio (WHR) ≥0.90 in men and ≥0.85 in women [23].

Lipodystrophy was defined as fat mass ratio (FMR) ≥1.5 [25].

Hypertension was defined as two consecutive systolic blood pressure (SBP) measurements ≥140 mmHg and/or diastolic blood pressure (DBP) measurements ≥90 mmHg, performed on different days (or) one single SBP measurement ≥140 mmHg and/or DBP measurement ≥90 mmHg, with the use of antihypertensives within 6 months of this measurement [13].

Metabolic syndrome was defined as the presence of three or more of the following factors: Waist circumference >94 cm in men and >80 cm in women, TGs ≥1.7 mmol/L, HDL cholesterol (HDL-c) <1.0 mmol/L in men and <1.3 mmol/L in women, SBP ≥130 or DBP ≥ 85 mmHg, or fasting glucose ≥5.6mmol/L [19].

Subclinical atherosclerosis was defined as an intima-media thickness (IMT) ≥0.9 mm at any site [24].

Discussion

Role of HIV Infection in the Pathogenesis of Atherosclerosis

**Figure 3:**
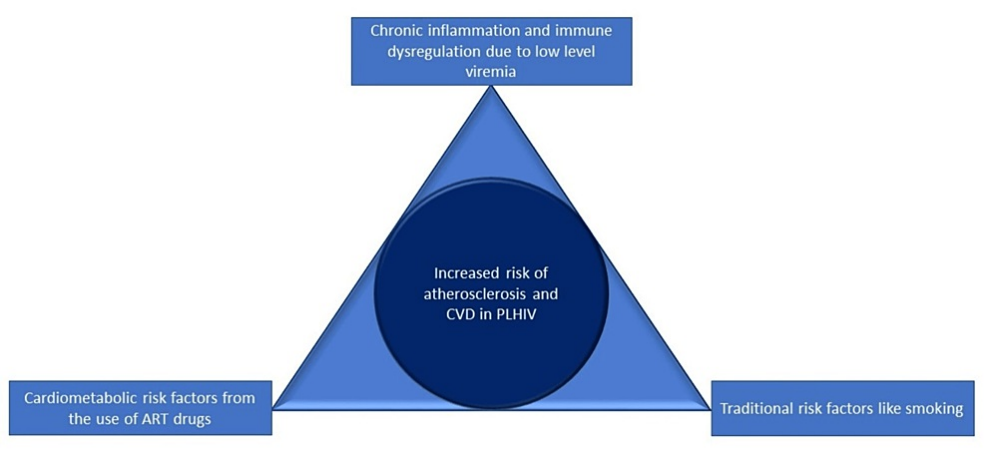
Contributors to CVD Risk in PLHIV ART: Antiretroviral therapy; CVD: cardiovascular disease; PLHIV: people living with HIV This figure is the author's own creation.

It has been established that HIV infection independently increases the risk for ASCVD [2-4]. This elevated risk is an amalgam of the greater burden of traditional cardiovascular risk factors in PLHIV [23], the side effects of antiretroviral drugs, and the chronic inflammation from HIV viremia [5,6] as depicted in Figure 3. Oxidized low-density lipoprotein (oxLDL), a biomarker of oxidative stress and inflammation, is purported to play a key role in atherogenesis [27] and has been linked to ASCVD [28]. oxLDL levels of the HIV-infected are higher compared to HIV-negative individuals [29] and positively correlate with the Framingham coronary heart disease risk score and carotid IMT (cIMT), a marker for subclinical atherosclerosis [30]. Statins help with lessening the levels of oxidative stress biomarkers, resulting in a reduced coronary plaque number independent of LDL levels [27,31].

Risk of Dyslipidemia

Dyslipidemia is the most prevalent cardiovascular risk factor in PLHIV contributing approximately 50% to the overall CVD risk [32,33]. The mechanisms professed to be involved in the pathogenesis of dyslipidemia in PLHIV are HIV-mediated chronic inflammation, immune activation [34,35], and ART-induced dyslipidemia [36-38]. Hypertriglyceridemia is the chief variety of ART-or HIV-related dyslipidemia [15]. In a retrospective cohort study by Zhang et al., in 2022, a PI-based regimen further bolstered the non-nucleoside reverse transcriptase inhibitor (NNRTI)-related hypertriglyceridemia in patients who switched from an NNRTI-based regimen. However, patients with normal TG levels just before the switch tend to sustain the normal levels of TGs [15]. In an analysis of the International Cohort Consortium of Infectious Disease (RESPOND) by Byonanebye et al., in 2021, dyslipidemia was less common with INSTI-based therapy than with PI-based therapy in both ART-naïve and ART-experienced individuals [16]. Other studies have also reported that INSTIs are less associated with the incidence of dyslipidemia than PIs [39-41], due to deterrence of endoplasmic reticulum stress [42]. INSTIs, therefore, remain the ART of choice in PLHIV owing to higher efficacy and safety. Rilpivirine (RPV), an NNRTI, had a lower risk of dyslipidemia than dolutegravir (DTG), an INSTI, while other INSTIs like elvitegravir/cobicistat (EVG/c) and raltegravir (RAL) had a greater risk than DTG [16]. Another retrospective cohort study by O’Halloran et al., in 2020, mentions no association between INSTIs and increased dyslipidemia risk [17]. A cross-sectional study by Bastard et al., in 2019, revealed an association between PI use and atherogenic dyslipidemia, but not LDL-related dyslipidemia [22]. All this evidence points to the need for follow-up, monitoring, and lifestyle counseling in patients taking ART, especially PIs.

Risk of Weight Gain and Obesity

A cohort study by Pantazis et al., in 2022, found that although the BMI levels increased in all ART groups, INSTIs were associated with acute BMI rise compared to NNRTIs [14]. The boosted PI group had a moderate rate of increase, almost comparable to that of the NNRTI group [14]. Further analysis of the INSTI group based on the core drug (DTG, EVG, or RAL) revealed that when compared to DTG or RAL, EVG was associated with piddling increases in BMI [14]. WHR was determinately higher with the NNRTI-based regimen of zidovudine (AZT)/emtricitabine (3TC)/nevirapine (NVP) compared with the boosted PI-based regimen of tenofovir disoproxil fumarate (TDF)/emtricitabine (3TC)/lopinavir-ritonavir (LPV/r) [23]. The retrospective cohort study by Bakal et al., in 2018, reinforced INSTI use as a prime risk factor for weight gain and obesity in PLHIV [18]. Individuals who are underweight at baseline have the greatest rise in BMI, with the plausible explanation of relatively superior immune reconstitution after ART initiation in individuals with more advanced HIV infection [43]. A steeper rise in weight or BMI in individuals receiving INSTI-based therapy has been substantiated in both clinical trials [44-47] and observational studies [18,48-52]. However, the mechanism of INSTI-associated weight gain remains unclear warranting further studies in this regard.

Risk of Metabolic Syndrome and Hypertension

In a cohort study by Byonanebye et al., in 2022, the incidence of hypertension with INSTI-based therapy was higher than with NNRTI-based therapy, but not different from PI-based therapy [13]. This was consistent with the findings of previous cohort studies [53-55]. The underlying mechanism though not clear yet, could be due to INSTI-associated metabolic derangements like weight gain, dyslipidemias, insulin resistance, adipogenesis, oxidative stress, and diabetes [13]. Although prior studies linked the use of older PIs to hypertension, this cohort showed a proportionately lower risk of hypertension, probably due to the use of newer PIs.

In the cross-sectional study by Hamooya et al., in 2021, participants receiving a DTG-based regimen had 2-fold higher odds of metabolic syndrome (MetS) than those receiving an NNRTI-based regimen [19]. The mechanisms through which INSTIs lead to MetS are currently little known, stressing the need for further research. Another cross-sectional study by Bastard et al., in 2019, depicted a positive correlation between diabetes and oxidative stress in PLHIV [22]. This justifies that oxidative stress, in addition to inflammation, could be a contributor to the greater burden of diabetes, insulin resistance, and MetS observed in PLHIV.

Risk of Lipodystrophy

In contrast to the findings by Domingo et al. [56], Martin et al. [57], and McComsey et al. [44], the cross-sectional study by Alikhani et al., in 2019, concluded that amassed exposure to RAL was autonomously associated with the elevated risk of lipodystrophy, as indicated by higher FMR values in these individuals [25]. This variation can be attributed to reverse causality: Prescription of RAL to highly experienced patients with heightened baseline levels of lipodystrophy.

Risk of CVD

Traditional risk factors like older age, male sex, smoking, hypertension, diabetes, and dyslipidemia increase the risk for ASCVD in the general population. In PLHIV, adverse effects of antiretroviral drugs may add to this. In an RCT by Llibre et al., in 2022, participants from the SWORD studies (SWORD-1 and SWORD-2) were tested for the biomarkers of inflammation and atherogenesis over 148 weeks [26]. Comparison of these values between the randomized continued antiretroviral regimen (CAR) and early-switch groups (CAR group: those who continued the three-drug or four-drug current antiretroviral regimen; early-switch group: those who switched to the two-drug regimen DTG+RPV) over 48 weeks revealed a decrease in fatty acid-binding protein-2 (FABP-2) levels favoring DTG+RPV [26]. In the non-comparative analysis of atherogenesis biomarkers through week 148, FABP-2 and soluble vascular cell adhesion molecule-1 (sVCAM-1) manifested a slight decline from the baseline values [26]. This study proves the non-inferiority of the two-drug regimen [26]. Through a retrospective analysis of insurance claims in the United States, Rebeiro et al., in 2023, concluded that the risk of certain cardiometabolic outcomes like MI, congestive heart failure (CHF), and lipid disorders is heightened in INSTI-based regimen users [12]. A cross-sectional study by Tiarukkitsagul et al., in 2021, observed that second-line ART (boosted PIs) was significantly associated with an intermediate to high 10-year ASCVD risk [20]. In a retrospective cohort study by O’Halloran et al., in 2020, when compared to non-INSTI-based regimens, INSTI-based therapy was found to have a 21% reduced risk of major adverse cardiac events (MACEs) including acute MI, stroke, coronary artery bypass grafting (CABG), and percutaneous coronary intervention (PCI) [17]. Most of the HIV-1-infected patients aged 50 years or older, from the cross-sectional study by Serrão et al., in 2019, had at least one non-AIDS-related comorbidity (NARC) with a mean of 2.1 [21]. The most prevalent NARC in this study population was hypercholesterolemia, followed by arterial hypertension [21]. Another cross-sectional study, by Calza et al., in 2019, recorded that cumulative exposure to PIs for > 5 years was greatly associated with an increased risk of subclinical atherosclerosis [24]. The lack of consistency on the risk of CVD with INSTIs in PLHIV should be addressed in order to frame evidence-based guidelines and appropriately dispense these drugs to patients with existing risk factors.

Early identification and management of these risks and NARCs among PLHIV, especially the aged PLHIV, is essential. However, we should be wary of polymedication and drug-drug interactions while treating these conditions. A comprehensive inclusion of the different fields of healthcare using a multidisciplinary approach is vital in cutting down the load of complex multi-morbid HIV infection in the elderly [21]. Whether the development and recent introduction of long injectable antiretroviral drugs addresses these issues remains to be seen.

Limitations

This systematic review is not without limitations. The articles selected for this review did not follow a uniform, standard tool/definition to assess the outcomes. For example, some of the studies on obesity only used BMI to diagnose obesity. There was a lack of standard parameters/measurement tools across the studies for assessing the risk of atherosclerosis. Certain studies had used cIMT, some had used common carotid artery (CCA)-IMT, and some had used ultrasonography to determine the number of plaques and plaques at high risk. The criteria for diagnosing diabetes, dyslipidemia, and other outcomes differed from one another. Further, only articles published within the last five years, with full text available for free were included. Studies that were not available in the English language, not in the database search, unpublished articles, reviews, editorials, and animal studies were all excluded. Studies with a sample population size of less than 100 were also excluded. Studies on patients with perinatally acquired HIV and a prior history of CVD were also excluded.

## Conclusions

Although the antiretrovirals curb the risk of atherosclerosis and CVD in PLHIV to a certain extent by reducing inflammation and boosting the immune system, they add to the CVD risk by altering the metabolic profile. In this review, we made an effort to ascertain the risk of CVD in PLHIV taking contemporary ART drugs. The order of risk of dyslipidemia among different drug classes is PIs>INSTIs>NNRTIs. Among INSTIs, the order is EVG/c and RAL>DTG. The order of risk for weight gain is INSTIs>PIs>NNRTIs. Among INSTIs, the order is DTG and RAL>EVG. The order of risk for hypertension is INSTIs and PIs>NNRTIs. The order of risk for MetS is INSTIs>NNRTIs. However, the risk of CVD associated with the specific drug classes varied in different studies. The healthcare providers must be mindful of these risks associated with specific ART drug classes along with the patients' individual risk factors when deciding on the optimal treatment plan or regimen for PLHIV. Routine assessment of CVD risk in individuals receiving ART can help with the timely intervention and prevention of adverse cardiac outcomes while simultaneously curtailing the economic burden in PLHIV.
